# Assessment of the Health Status of the Oldest Olds Living on the Greek Island of Ikaria: A Population Based-Study in a Blue Zone

**DOI:** 10.1155/2019/8194310

**Published:** 2019-11-30

**Authors:** Romain Legrand, Patrick Manckoundia, Gilles Nuemi, Michel Poulain

**Affiliations:** ^1^Department of Geriatrics and Internal Medicine, Hospital of Champmaillot, University Hospital of Dijon Bourgogne, Dijon, France; ^2^INSERM U-1093, Cognition, Action and Sensorimotor Plasticity, University of Burgundy Franche-Comté, Dijon, France; ^3^Department of Biostatistics and Bioinformatics, François Mitterrand Hospital, University Hospital of Dijon Bourgogne, Dijon, France; ^4^Institute for the Analysis of Change in Historical and Contemporary Societies (IACCHOS), Université Catholique de Louvain, Belgium; ^5^Estonian Institute for Population Studies, Tallinn University, Estonia

## Abstract

**Objective:**

To describe the demographic characteristics, socio-economic status, functional status (autonomy, strength), and health status (cognitive and thymic functions, cardiovascular risk factors, and nutritional status) of the oldest olds living on the Greek island of Ikaria. We also try to explain the longevity observed in this population.

**Methods:**

A cross-sectional observational study of people aged 90 and over living in both municipalities of north-western Ikaria (Evdilos and Raches) was conducted over one year, from October 21, 2012 to October 21, 2013. The participants were interviewed (medical history), had a brief clinical examination, and underwent standardized geriatric assessments including the Geriatric Depression Scale (GDS-15), the Mini-Mental-State Examination (MMSE), the Activities of Daily Living (ADL), the Instrumental ADL (IADL), and an assessment of grip strength.

**Results:**

Seventy-one persons (37 females, 34 males), aged 94.1 years on average, were interviewed at their homes. Seven percent were current smokers (females 5.4%, males 8.8%). Hypertension was diagnosed in 70.4% of participants, diabetes in 19.7%, hypercholesterolemia in 12.7%, and obesity in 17.2%; 66.0% of the population had one chronic disease or more. The mean score for the GDS-15 scale was 3.7/15.0, 23.7/30.0 for the MMSE, 4.0/6.0 for the ADL, and 4.2/8.0 in females and 3.6/5.0 in males for the IADL. Grip strength was 17.0 kg in females and 26.5 kg in males.

**Conclusions:**

This study provides an overview of the socio-demographic and medical characteristics of the oldest olds living in a longevity Blue Zone.

## 1. Introduction

As the “baby boom” generation has gotten older, life expectancy has increased considerably [1]. Considering that people aged ≥80 face the highest rates of disability and dementia, an increase in this population is a major burden for public health [2, 3]. The most elderly demographic, known as the oldest olds (OOs), often suffer from frailty syndrome, which is associated with functional decline and increased mortality [4]. With advancing age, people tend to develop multiple chronic diseases that negatively affect function and quality of life [5]. On the contrary, successful aging refers to a scenario in which functional capacity is maintained and individuals are free from disease [6].

Most studies that look at aging have focused on the younger elderly and excluded the OOs, who remain poorly understood. With a focus on these OOs, some studies have identified regions with exceptional longevity that are now referred to as Longevity Blue Zones (LBZ) [7, 8]. In practice, a LBZ is defined as a relatively limited and homogenous geographical area where the population shares the same lifestyle and environment, and the longevity of the population is exceptionally high. Validated LBZs are found, for instance, in Okinawa (Japan), on the Nicoya peninsula (Costa Rica) and on the island of Ikaria (Greece) [8].

The aim of our study was to assess health status of people aged ≥90 living in the northwestern part of Ikaria and, in doing so, to contribute to a better understanding of their exceptional longevity.

## 2. Methods

### 2.1. Study Design

From 21/10/2012 to 21/10/2013, we conducted a cross-sectional observational study on the health status of people aged ≥90 living in two municipal units, Evdilos and Raches, located in the northwestern part Ikaria. Longevity was found to be higher in these two municipalities than in the southern municipality of Agios Kirikos, as reported by Poulain & Pes [9].

### 2.2. Population

Participants were identified using the lists established by Poulain and Pes in 2009. We updated these lists by consulting the local population registers (Dimotologio) that were available in the municipal offices of Evdilos and Raches and by visiting each village in these municipal units. The list of potential participants aged ≥90 was completed by involving participants from the survey of Panagiotakos et al. [9, 10]. Finally, some data were also provided by the healthcare centres in Evdilos and Raches.

This study was conducted in accordance with the Declaration of Helsinki and national standards. Approval from an ethics committee was not necessary for this type of simple survey among voluntary participants and with no modification to usual care.

### 2.3. Questionnaire

The questionnaire was divided into several parts. Socio-economic data were collected: (1) Identity: name, birth date, place of birth, and father's name (to distinguish potential namesakes); (2) Location of main and secondary residences. For the main residence, participants defined their main home as: isolated; within a village; or within a nursing home; (3) Number of years living in Ikaria; (4) Educational level: abilities in reading, writing and calculation; (5) School: primary, secondary or higher; (6) Profession before retirement; (7) Monthly income and source: personal, family or state; (8) Family status: single, widow(er), cohabitant, married or divorced; (9) Family environment: number of siblings, number of children, number of people living in the home of the participant; (10) Habits: current or past smoking with the year of cessation, equivalent alcohol consumption in glasses of wine per day.

The following medical data were recorded: (1) Self-rated health (SRH) measured with a 5-point Likert scale (very good, good, medium, poor or very poor); (2) Self-evaluation of sleep quality measured with a 5-point Likert scale and duration; (3) Medical history: cardiovascular (CV) disease or risk factors or previous management, and certain predefined diseases common in OOs (chronic obstructive pulmonary disease (COPD), cancer, osteoporosis, dysthyroidism); (4) Current medication; (5) Clinical examination with measurement of weight, height, systolic blood pressure (BP), diastolic BP and resting heart rate. Hypertension was defined by systolic BP ≥140.0 mm Hg and/or diastolic BP ≥90.0 mm Hg and/or a prior diagnosis of hypertension and/or antihypertensive treatment [11].

A standardized geriatric assessment was performed, including: (1) mood assessment using the 15-item Geriatric Depression Scale (GDS-15) [12]; (2) cognitive assessment using the Mini-Mental-State Examination (MMSE) [13]. The MMSE was only used in subjects able to read, write, calculate, see and hear, without a history of depression or stroke. Thus, some illiterate individuals were excluded. Because the MMSE scores are influenced by several factors including age, educational level, and acute disease [14, 15], we preferred to calculate only the average of the MMSE score, its extreme values and the proportion of subjects with MMSE score ≥24.0, without classifying people by cognitive impairment (absent, mild, moderate or severe); (3) functional assessment using the Activities of Daily Living (ADL) scale scored out of 6.0 and the Instrumental ADL (IADL) scale scored out of 8.0 in females and 5.0 in males [16, 17]; and (4) a nutritional assessment using body mass index (BMI).

Grip strength (GS) was also measured. The measurement was repeated three times for each hand, and the best result was retained [18].

### 2.4. Statistical Analysis

Continuous variables were expressed as means, medians and interquartile ranges, and categorical variables as numbers and percentages.

At first, calculations were done using data from the entire population and then the data stratified by gender when there was a contrast in prevalence between females and males.

Women and men were compared in terms of age, educational level, socio-professional category, income, marital status, smoking, alcohol consumption, SRH, medical history, current medication, BP, heart rate, GDS, ADL and IADL scores, BMI, and GS.

Frequencies were compared using the chi-2 test or Fisher's exact test when appropriate. Means were compared using the Student's unpaired t-test or the Wilcoxon rank sum test according to the result of the normality test. Normality was assessed using the Shapiro-Wilk test.

SAS 9.3 software was used to conduct all statistical analyses. All statistical tests were two sided.

Statistical significance was set at *P* < 0.05.

## 3. Results

Among the 98 eligible participants, 71 were enrolled. Twenty-one were not seen because they were temporarily overseas, 1 refused to take part and 5 died while the surveys were being conducted.

The 71 participants were aged 94.1 ± 3.4 on average (median 93.0). There were 37 females (F) and 34 males (M) (Table 1).

Of the 27 eligible persons who did not participate in the study, 18 were females and 9 were males. Their mean age was 94.8 ± 3.8 (95% confidence interval (CI): 93.4–96.2). There was no significant difference in age (*P* = 0.380) or gender (*P* = 0.285) between participants and nonparticipants.

### 3.1. Socio-Demographic and Economic Characteristics (Table 2)

The 71 participants were interviewed and examined at their place of residence. The help of a proxy was necessary for 6 females and 2 males. Most participants (95.7%) were born in Ikaria and had always lived there. About half of the population lived in a village and the other half in an isolated house. One female lived in a nursing home.

The average number of school years was 6.6 ± 2.9 (primary school). The majority had learned to read, write, and calculate. The most common occupation was farming. The average monthly income was 611.4 ± 444.8€; the median was 500.0€ (range 200.0–2500.0).

Most women were widowed, whereas more than half of the men were still married. On average, participants had 5.5 ± 2.6 siblings and 3.3 ± 1.8 children. Regarding the size of the household, 52.2% of participants lived in a household of two, 23.9% more than two and 23.9% alone. Among those who lived alone, there were more females (27.0%) than males (20.6%).

More than half of the population was nonsmoking, but most of the men had smoked at some point and almost 10% still smoked. The average age of smoking cessation was 60.6 ± 19.0 years. About 75% of the population drank alcohol regularly, usually 1–2 glasses a day. Alcohol consumption was more common in men than in women (*P* = 0.004).

### 3.2. Medical Data

For SRH and sleep quality, the “good” and “very good” categories and the “poor” and “very poor” categories were grouped together because of the small number of participants in the two “very” categories. SRH and sleep quality were “very good” to “good” for more than half of the population, especially in males. Mean sleep duration was 9.0 ± 1.8 hours; 8.8 ± 1.7 hours in females and 9.1 ± 1.8 hours in males, with no significant difference between the 2 genders (*P* = 0.473). Mean systolic BP was 133 ± 17.4 mm Hg and mean diastolic BP was 70.7 ± 9.7 mm Hg for the entire population. The mean heart rate was 68.6 ± 10.6/min. Hypertension was found in 70.4% of participants, diabetes in 19.7% and dyslipidemia in 12.7%, with no significant difference between men and women. At least one CV disease had been diagnosed in 38.0% of participants (40.5% of women, 35.3% of men). There was no difference between females and males for CV disease or other comorbidities apart from COPD, which was more prevalent in males than in females. Overall, 81.7% of participants were taking a medical treatment, with significantly more females (91.9%) than males (70.6%) (*P* = 0.020) (Table 3). In addition, 23.9% of participants were taking more than three medications daily.

### 3.3. Standardized Geriatric Assessment

The mean GDS-15 score was 3.7 ± 3.8. It was significantly higher among females (4.2 ± 4.1) than in males (3.2 ± 3.5). In total, 11.8% of the total population (17.1% of females and 6.1% of males) had a GDS-15 score ≥10.0. The mean MMSE score, obtained in 46 subjects (20 females and 26 males), was 23.7 ± 5.0 (95% CI: 22.3–25.1) (range 11–30), and 39.1% of the population may have had cognitive impairment (60.0% of females and 23.1% of males). An ADL score ≥5.0 was obtained in 59.5% of females and 88.2% of males. The IADL scores indicated that 51.4% of females and 61.8% of males had a high level of autonomy. Mean BMI was 26.2 ± 3.6 kg/m^2^ in the whole population. Only 42.2% were normal weight, while 40.6% were overweight and 17.2% were obese. Mean GS was 17.0 ± 6.7 kg in women (median 20.0) and 26.5 ± 8.9 kg in men (median 25.0). Except for MMSE scores, which were significantly higher in females than in males, and GS, which was significantly lower in females than in males, there was no significant difference between genders for the other parameters (GDS-15, ADL, IADL and BMI) (Table 4).

## 4. Discussion

One of the main strengths of this study is the fact that our investigator went in person to the participants' homes, and so our information was not based on questionnaires sent by post or email. We were therefore able to get a closer look at the participants' way of life. Our survey adds to the existing knowledge regarding the OOs living in a LBZ by providing novel data such as GS.

The studied population was representative of the officially recognized population of OOs in the municipal units of Raches and Evdilos. The socio-economic status of this population was low. We found that depression was infrequent (11.8% of the total population; 17.1% of women and 6.1% of men). Although it is difficult to specify the levels of dementia, functional abilities such as muscle strength were good. CV risk factors such as smoking, hypertension, diabetes, and overweight were significant. Multimorbidity was low, as was polypharmacy. The SRH was good or very good for more than half of females and almost 70% of males in the studied population.

Although eligible for this study, 27 of the OOs did not participate, mostly because they were temporarily overseas. Because no statistical difference was found between the group that was included and the group that was not, the study population was representative of the officially recognized population.

The data regarding income, education, and occupation revealed a low socio-economic status overall, suggesting that the longevity of this population is independent of socio-economic conditions. This finding is interesting because we know that there is a social gradient in health, but it should be interpreted with caution because the correlation between income and longevity in Ikaria was not assessed. It is possible that the low income of the OOs is only a reflection of the overall low income of the inhabitants of Ikaria. Socio-economic status was also found to be low in other LBZ like the Nicoya region of Costa Rica and Okinawa in Japan [19, 20]. The absence of a social gradient on the Nicoya peninsula has been explained by its public health insurance system which is almost universal and its network of primary healthcare providers [20]. Cockerham & Yamori explained it in Okinawa by its healthy lifestyles, especially diet, and the social support of family and friends [19]. Poor social relations, negative labour market experiences like unemployment, and poor health behaviour are suggested explanations for the socio-economic gradient in health [21]. In Ikaria, we believe that the study population has remained relatively unaffected by these factors.

Mood assessment with the GDS-15 showed results similar to those found on the island in 2009 [10]. Our results confirm a low prevalence of depression in the OOs living in the Ikaria LBZ. The GDS-15 scores were slightly higher in females than in males, suggesting that females had more depressive tendencies than males, which is consistent with previous studies of depression [22, 23]. Chrysohoou et al. found that fish consumption moderates the symptoms of depression among the elderly in Ikaria [24]. Good social relationships may also play a role [25].

Based on the 24/30 threshold, our survey suggests that 39.1% of the population may have cognitive impairment. MMSE scores were worse in females than in males, which is consistent with previous studies assessing cognitive impairment [3]. However, they do not allow the diagnosis of dementia, which is cognitive impairment that interferes with autonomy and the ability to perform usual activities. Dementia can only be diagnosed after a consistent assessment including neuropsychological tests [26].

We found that 73.2% of the population had high ADL scores and 56.3% had high IADL scores, which means that the majority had maintained their ability to carry out their usual activities. Once again, the scores were higher in men, which is consistent with previous studies [2, 27].

GS is recognized as a key objective measurement of overall muscle strength, and it is used in clinical practice to assess frailty and sarcopenia [28, 29]. It is a predictor of functional limitations, disability and mortality [30]. One study highlighted the existence of a north-south gradient of diminishing GS [31]. However, the OOs of our survey had a higher mean GS (17.4 for women; 27.3 for men) than their Italian (9.2 for women; 14.2 for men) and Danish counterparts (12.2 for women; 24.2 for men). These results suggest that the OOs living in the Ikaria LBZ are particularly strong, which is associated with good functional abilities. Another criterion used to assess frailty according to Fried is malnutrition [28]. Though no one was underweight in the study population, it is interesting to note that a significant proportion (40.6%) was overweight. Indeed, some authors have highlighted a U-shaped relationship between BMI and the risk of developing major mobility issues, and the risk for individuals with a BMI of 25.0-30.0 was half of that for those with a BMI less than 25.0 or more than 30.0 [32]. Perhaps the large number of overweight participants found in the Ikaria LBZ is connected to their particularly high muscle strength, seeing as sarcopenia is related to the loss of muscle strength [29].

SRH is an indicator of successful aging that represents the ability of an individual to adapt to changes in health [33]. In most of our participants, especially men, both SRH and sleep quality were good. Aging is often associated with a decrease in sleep quality, which leads to the use of sedatives or hypnotics to improve sleep, but which in turn can cause other side effects [34]. In our population, issues with sleep were more common in women, as in other studies among the OOs [35]. The use of psychoactive medications to induce sleep was higher in women, but globally lower in our population compared with others [35]. The good sleep quality observed in Ikaria's OOs could be explained by a low level of depression, good SRH, and the contribution of a daily routine with natural exposure to sunlight [36]. Moreover, the mean sleep duration found in our population has been associated with a lower risk of all-cause mortality, especially CV-related mortality [37].

Women were significantly less likely than men to smoke or to drink alcohol. This is in accordance with the literature for this generation [38]. Though many men had smoked, most of them had stopped. However, the proportion of active smokers in our population was higher than in the OOs elsewhere in Greece (8.0 versus 2.2%) and, especially among men (12.0 versus 1.1%) [39]. In our study, approximately two-thirds of participants, especially men (90.0%), drank moderate amounts of alcohol regularly. Epidemiologic studies have shown a U-shaped relationship between alcohol consumption and all-cause mortality in that moderate consumers of alcohol have a reduced risk of all-cause mortality in comparison with nondrinkers and heavy drinkers [40]. The protective effect exists for drinkers of wine, like in Ikaria, and it persists in elderly people [41, 42].

Our study showed a high prevalence of hypertension (70.4% of participants) in the OOs of Ikaria, mainly among women. This result is similar to that found by Psaltopoulou et al., who showed a higher prevalence of hypertension among Greek women than in other European women (Psaltopoulou, Orfanos, Naska, Lenas, Trichopoulos, & Trichopoulou, 2004). However, despite this high prevalence, most of our subjects were taking antihypertensive medication and had normal BP. Treatment of hypertension in OOs has shown its effectiveness for stroke and heart failure prevention and for reducing all-cause mortality [43]. It also shows that the OOs living in the Ikaria LBZ have adequate access to health care.

Aging is associated with an increase in the number of diseases (referred to as multimorbidity) with an impact on quality of life, independency, and healthcare costs [5]. On Ikaria, we found that 37.5% of the OOs were free from disease, compared with 21.0% in Greece and 26.0% in France [39]. It may seem surprising given the high prevalence of some CV risk factors on Ikaria. Similar results were found by the Seven Countries Study Research Group where the absolute risk of death from coronary heart disease at similar BP and serum cholesterol levels was lower in Mediterranean countries, which the authors attributed to the Mediterranean diet [44].

Multimorbidity often involves the use of multiple medications, referred to as polypharmacy, which in turn increases the risk of geriatric syndromes, morbidity, and mortality [45]. There is not a consistent cut-off to define polypharmacy. On Ikaria, although 81.7% of the OOs were taking at least one drug per day, only 23.9% were taking more than three medications daily. This is much less than in most studies on the elderly, where about half take more than five medications daily [45]. The low prevalence of polypharmacy observed on Ikaria may be due to the low prevalence of multimorbidity.

Overall, our results suggest that the OOs in Ikaria have a good health status. Indeed, though hypertension was common, the rate of depression was low, as was the number of participants who consumed more than three medications daily. In addition, the majority of participants rated their general health as good. In comparison, the Umea 85+ Study, a survey conducted in Sweden on the health status of OOs, showed that over half of the participants had hypertension, a quarter was depressed and the mean number of drugs taken was 6.4 ± 4.0 [46]. Given the results of our study, we can conclude that males were generally healthier than females. This is in accordance with other European studies on OOs that made similar observations [47].

We recognize that our study has some limitations. First, the number of included participants was relatively small. In addition, though our study was population-based, some OOs in the target area could not be found, creating a potential selection bias.

In our study, some data were self-reported, which may create an information bias. For example, the collection of data on diseases may have generated an underestimation of their prevalence.

Another limitation is the lack of diet evaluation. We would have liked to include a diet questionnaire and/or the Mini Nutritional Assessment (MNA). However, we chose not to overload the survey in order to encourage a high participation rate, which can be a challenge in such an elderly population. In addition, self-reported food-related responses regarding quality and quantity would have been difficult to verify without daily visits from an investigator. We therefore preferred to limit our analysis of nutritional state to measuring the weight and calculating the BMI.

## 5. Conclusion

In conclusion, our study suggests that the OOs from the Ikaria LBZ have aged well. Indeed, their functional abilities were high, they had few medical conditions and they perceived their health as good or very good. It would be relevant to compare the OOs of Ikaria with those of other LBZ in order to identify possible pathways to longevity.

## Figures and Tables

**Table 1:** Characteristics of the oldest olds included in the study according to age and gender.

Age	Females	Males	Total
90	5	5	10
91	6	5	11
92	4	6	10
93	3	3	6
94	1	5	6
95	2	1	3
96	2	1	3
97	6	3	9
98	1	1	2
99	3	2	5
100	1	2	3
101	2	0	2
102	1	0	1
*Total*	37	34	71

**Table 2:** Socio-demographic characteristics of participants.

Parameter		Females	Males	Total	*P* ^∗^
		*N* = 37	*N* = 34	*N* = 71	
		*n* (%) or mean ± SD	*n* (%) or mean ± SD	*n* (%) or mean ± SD	
Place of birth	Ikaria	33 (94.3)	32 (94.1)	65 (94.2)	0.976
	Other	2 (5.7)	2 (5.9)	4 (5.8)	

Residence	House in a village	20 (54.1)	21 (61.8)	41 (57.7)	0.546
	Isolated house	16 (43.2)	13 (38.2)	29 (40.8)	
	Nursing home	2.7 (1)	0 (0.0)	1 (1.5)	

Years of school		6.2 ± 2.4	7.1 ± 3.2	6.6 ± 2.9	0.182

Occupation	Farmers	12 (32.4)	9 (26.5)	21 (29.6)	*<0.001*
	Housewives	15 (40.5)	0 (0)	15 (21.1)	
	Sailors, Fishermen	0 (0.0)	8 (23.5)	8 (11.3)	
	Workers	0 (0.0)	6 (17.6)	6 (8.4)	
	Intermediate	3 (8.1)	3 (8.8)	6 (8.4)	
	Craftsmen, Trader	4 (10.8)	2 (5.9)	6 (8.4)	
	Coal miner	0 (0.0)	4 (11.8)	4 (5.6)	
	Other	3 (8.1)	2 (5.9)	5 (7.0)	

Income (€)		553.9 ± 410.6	670.7 ± 476.9	611.4 ± 444.8	0.271

Marital status	Widowed	30 (81.1)	14 (41.2)	44 (62.0)	*<0.001*
	Married	4 (10.8)	20 (58.8)	24 (33.8)	
	Other	3 (8.1)	0 (0.0)	3 (4.2)	

Family	Siblings	5.8 ± 2.8	5.1 ± 2.5	5.5 ± 2.6	0.272
	Children	3.7 ± 1.9	2.8 ± 1.6	3.3 ± 1.8	*0.04*

Tobacco	Nonsmoker	35 (94.6)	5 (14.7)	40 (56.3)	*<0.001*
	Former smoker	0 (0.0)	26 (76.5)	26 (36.6)	
	Current smoker	2 (5.4)	3 (8.8)	5 (7.1)	

Alcohol (glasses/day)	No alcohol	16 (43.2)	3 (8.8)	19 (26.8)	*0.004*
	1–2	19 (51.4)	26 (76.5)	45 (63.4)	
	≥3	2 (5.4)	5 (14.7)	7 (9.8)	

*N*: number of subjects of group population, *n*: number of subjects for each category parameter, SD: standard deviation.

^∗^Chi-2 test or Fisher's exact test for frequencies and Student's unpaired *t*-test for means were used.

**Table 3:** Medical data of participants.

Parameter		Females	Males	Total	*P* ^∗^
		*n/N* (%) or mean ± SD (*N*)	*n/N* (%) or mean ± SD (*N*)	*n/N* (%) or mean ± SD (*N*)	
Self-rated health	Very good to good	18/34 (52.9)	23/33 (69.7)	41/67 (61.2)	0.273
	Medium	12/34 (35.3)	6/33 (18.2)	18/67 (26.9)	
	Very poor to poor	4/34 (11.8)	4/33 (12.1)	8/67 (11.9)	

Sleep	Mean duration	8.8 ± 1.7	9.1 ± 1.8	9.0 ± 1.8	0.473
	Very good to good quality	18/33 (54.5)	20/34 (58.8)	38/67 (56.7)	0.813
	Acceptable quality	12/33 (36.4)	10/34 (29.4)	22/67 (32.8)	
	Very bad to bad quality	3/33 (9.1)	4/34 (11.8)	7/67 (10.5)	

BP (mmHg)	SBP	130.7 ± 16.6 (36)	135.6 ± 18.1 (32)	133.0 ± 17.4 (68)	0.238
	DBP	70.8 ± 9.5 (36)	70.6 ± 10.0 (32)	70.7 ± 9.7 (68)	0.931

Heart rate (min)		66.4 ± 9.0 (28)	70.7 ± 11.7 (31)	68.6 ± 10.6 (59)	0.086

Cardiovascular risk factors	Hypertension	28/37 (75.7)	22/34 (64.7)	50/71 (70.4)	0.314
	Diabetes	8/37 (21.6)	6/34 (17.6)	14/71 (19.7)	0.674
	Dyslipidemia	5/37 (13.5)	4/34 (11.8)	9/71 (12.7)	0.839

Comorbidities	Cardiovascular diseases	15/37 (40.5)	12/34 (35.3)	27/71 (38.0)	0.660
	COPD	1/37 (2.7)	6/31 (19.4)	7/68 (10.3)	*0.034*
	Cancer	2/37 (5.4)	3/31 (9.7)	5/68 (7.4)	0.543
	Osteoporosis	5/37 (13.5)	2/31 (6.5)	7/68 (10.3)	0.379
	Dysthyroidism	8/37 (21.6)	2/31 (6.5)	10/68 (14.7)	0.091

Drugs	All drugs	34/37 (91.9)	24/34 (70.6)	58/71 (81.7)	*0.020*
	Cardiovascular drugs	30/37 (81.1)	21/34 (61.8)	51/71 (71.8)	0.080
	Psychotropic drugs	12/37 (32.4)	8/34 (23.5)	20/71 (28.2)	0.422

*n*: number of subjects for each category of the considered parameter, *N*: number of subjects who were assessed for each parameter in each group (females, males or total), SD: standard deviation, BP: blood pressure, SBP: systolic blood pressure, DBP: diastolic blood pressure, COPD: chronic obstructive pulmonary disease.

^∗^Chi-2 test or Fisher's exact test for frequencies and Student's unpaired *t*-test for means were used.

**Table 4:** Standardized geriatric assessment of participants.

Parameter		Females	Males	Total	*P* ^∗^
		*n/N* (%) or mean ± SD (*N*)	*n/N (%)* or mean ± SD (*N*)	*n/N (%)* or mean ± SD (*N*)	
GDS-15	<5	22/35 (62.9)	25/33 (75.8)	47/68 (69.1)	0.331
	≥10	6/35 (17.1)	2/33 (6.1)	8/68 (11.8)	

MMSE (/30)	≥24	8/20 (40.0)	20/26 (76.9)	28/46 (60.9)	*0.011*
	Mean ± SD (N)	21.4 ± 5.6 (20)	25.5 ± 3.6 (26)	23.7 ± 5.0 (46)	*<0.001*

ADL (/6)	≥5	22/37 (59.5)	30/34 (88.2)	52/71 (73.2)	0.053
	<3	10/37 (27.0)	4/34 (11.8)	14/71 (19.7)	

IADL	High	19/37 (51.4)	21/34 (61.8)	40/71 (56.3)	0.358
	Medium	6/37 (16.2)	7/34 (20.6)	13/71 (18.3)	
	Low	12/37 (32.4)	6/34 (17.6)	18/71 (25.4)	

BMI (kg/m^2^)	<18.5	0 (0.0)	0 (0.0)	0 (0.0)	0.896
	18.5–24.9	13/33 (39.4)	14/31 (45.2)	27/64 (42.2)	
	25–29.9	14/33 (42.4)	12/31 (38.7)	26/64 (40.6)	
	≥30	2 6/33 (18.)	5/31 (16.1)	11/64 (17.2)	

Grip strength (kg)		17.0 ± 6.7 (28)	26.5 ± 8.9 (33)	22.1 ± 9.3 (61)	*<0.001*

*n*: number of subjects for each category of the considered parameter, *N*: number of subjects who were assessed for each parameter in each group (females, males or total), SD: standard deviation, GDS-15: geriatric-depression-scale-15 items, MMSE: mini-mental-state-examination, ADL: activities of daily living, IADL: instrumental activities of daily living, BMI: body mass index.

^∗^Chi-2 test or Fisher's exact test for frequencies and Student's unpaired *t*-test for means were used.

## Data Availability

Our data are available and can be provided if needed.
